# Breeding phenology and winter activity predict subsequent breeding success in a trans-global migratory seabird

**DOI:** 10.1098/rsbl.2015.0671

**Published:** 2015-10

**Authors:** A. Shoji, S. Aris-Brosou, A. Culina, A. Fayet, H. Kirk, O. Padget, I. Juarez-Martinez, D. Boyle, T. Nakata, C. M. Perrins, T. Guilford

**Affiliations:** 1 Department of Zoology, University of Oxford, South Parks Road, Oxford OX1 3PS, UK; 2 Department of Mathematics and Statistics, University of Ottawa, Ottawa, Canada K1N 6N5

**Keywords:** phenology, migration, machine learning, adaptive boosting, multi-event capture–mark–recapture model

## Abstract

Inter-seasonal events are believed to connect and affect reproductive performance (RP) in animals. However, much remains unknown about such carry-over effects (COEs), in particular how behaviour patterns during highly mobile life-history stages, such as migration, affect RP. To address this question, we measured at-sea behaviour in a long-lived migratory seabird, the Manx shearwater (*Puffinus puffinus*) and obtained data for individual migration cycles over 5 years, by tracking with geolocator/immersion loggers, along with 6 years of RP data. We found that individual breeding and non-breeding phenology correlated with subsequent RP, with birds hyperactive during winter more likely to fail to reproduce. Furthermore, parental investment during one year influenced breeding success during the next, a COE reflecting the trade-off between current and future RP. Our results suggest that different life-history stages interact to influence RP in the next breeding season, so that behaviour patterns during winter may be important determinants of variation in subsequent fitness among individuals.

## 1. Introduction

Individual variation in both quality and reproductive performance (RP) offers critical insights into evolutionary ecology. While conventional theories in vertebrates propose that such variation is mostly owing to age or experience, recent work suggests that some individuals perform better regardless of their age (e.g. [1]). In particular, carry-over effects (events in one season affecting performance in the next; COEs) are recognized as critical to individual RP (e.g. [2,3]). However, it is difficult to quantify the conditions faced by highly mobile migratory animals during migration itself or over winter. Seabirds disperse on the open ocean and may migrate long distances during the non-breeding season, making them good candidates for addressing how (if at all) COEs impact RP, because the migratory stage can constitute most of their annual life cycle. Most research on individual COEs has therefore focused on non-time-series data such as wintering locations [4,5], phenology [6], diet [7], hormone levels [8] or body condition [9]. How the interaction between phenology and wintering behaviour affects RP has only recently started to be explored [10,11]. We approached this outstanding issue in a long distance migratory seabird, the Manx shearwater (*Puffinus puffinus*), combining direct observations of RP with a computational approach that monitors year-round daily activity, over a 5-year period. We show how both the timing of events in the annual cycle and winter at-sea behaviour help to predict RP.

## 2. Material and methods

The study was conducted on birds breeding on Skomer Island, UK (51°44′ N, 5°17′ W), the world's largest Manx Shearwater colony [12], between 2009 and 2014. Study burrows were visited daily during breeding seasons to record breeding progress directly. Geolocators (Biotrack Ltd: 20 × 9 × 5.5 mm; mass 1.5–2.4 g, approximately 0.3% of mean 450 g body mass), with saltwater-immersion logging capability, were deployed on and recovered from adult individuals, attached with custom-made darvic leg-rings, resulting in five consecutive years of recorded migratory behaviour (2009–2014). We included only experienced breeders that had raised young prior to the first observation period (electronic supplementary material, table S1).

At-sea behaviour, from date of colony departure to date of return, and timing of events (phenology), were analysed to examine whether these could predict breeding performance. At-sea behaviour and timing of events during migration and winter were determined in two ways. First, saltwater-immersion data (SID) were used to quantify the proportion of time in flight ([13]; electronic supplementary material) during daytime, to determine how individual flying activity increased or decreased over the course of each winter. Breakpoints in the patterns of activity were found by performing piecewise linear regressions for proportion of flight time versus date; breakpoints (number and positions) were determined by the bootstrap restarting algorithm implemented in the segmented R library [14]. Second, these flight phenologies were then validated by comparing the phenologies against daily positions derived from light loggers [15]. Similar analyses were performed for resting and foraging behaviours—for each bird during each year.

To assess predictive power of phenology on individual RP, we employed a supervised machine-learning algorithm based on adaptive boosting [16], which is essentially combining weakly informative features (e.g. fledging dates) into a strong predictor of RP (electronic supplementary material). To understand the biological nature of this predictive power, we analysed behavioural patterns based on SID. For this, time-series additive decompositions were performed on individual tracks; nonlinear trends were then averaged by RP (success, fail or skip; electronic supplementary material) and by activity type (rest, fly, forage; electronic supplementary material); these trends were finally summarized by their empirical cumulative distributions to reveal associations between RP and activity during wintering. To understand the long-term viability of such associations, we implemented a multi-event capture–mark–recapture (MECMR) model. MECMR summarized year-to-year reproductive data as a transition matrix giving the frequencies of RP state changes from one year to the next. For this last step, sample size was expanded to include 88 additional individuals (41 females) without Geolocator data but that were nonetheless monitored for breeding progress. The approach also allowed us to test for individual heterogeneity with a mixture model (electronic supplementary material).

## 3. Results

Geolocator data were successfully retrieved for 111 bird-seasons: 64 from birds that raised a chick, 29 that failed during incubation and 18 that failed to lay in the season preceding the tracked winter (electronic supplementary material, table S1). Each bird-season consisted of two consecutive seasons of breeding phenology (pre- and post-migration) as well as migration phenology. Overall study impact was assessed by comparing the number of chicks per egg laid by study birds (0.86 ± 0.13 s.d.) with that in an adjacent unmanipulated plot (0.63 ± 0.07 s.d.; [17]), indicating that there was no measurable negative effect on reproduction (see details in the electronic supplementary material).

### (a) Adaptive boosting extracts reproductive performance information from phenology

**Figure 1.**
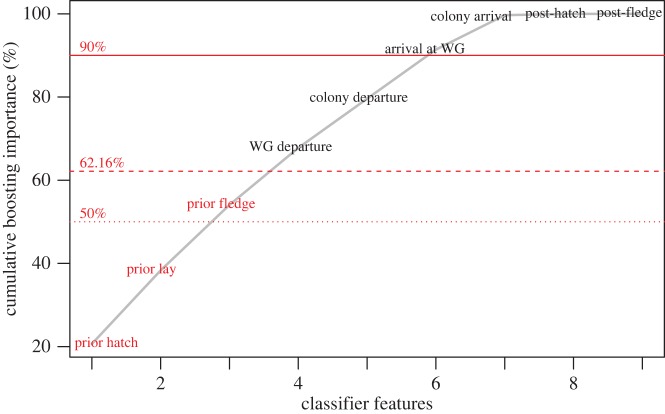
Ranking of classifier features according to their cumulative importance. The SAMME algorithm [18] gave most importance to prior events (in red). The 10× cross-validation success rate of the classifier is 62.16%; all features above this value (in black) have negligible predictive power with respect to RP. WG, wintering grounds.

With the classifier trained on the whole dataset, we found that hatch and lay dates in the previous year were the two most important features used for classification, explaining approximately 40% of the information extracted (figure 1). The 10-fold cross-validation error rate was 37.84% (electronic supplementary material, table S2), smaller than expected by chance, indicating that our model had satisfactory predictive power (*p* < 0.0001; electronic supplementary material, figure S1) even if it predicts breeding success better than failure. Eliminating the most correlated variables (prior hatch and colony departure and arrival dates: electronic supplementary material, figure S2) slightly increased classification error rate to 39.00%, so all features were kept in the final classifier. Thus a combination of all three prior events—hatch date, lay date and fledge date—offers the strongest predictor of RP (figure 1).

### (b) Winter behaviour versus breeding performance

**Figure 2.**
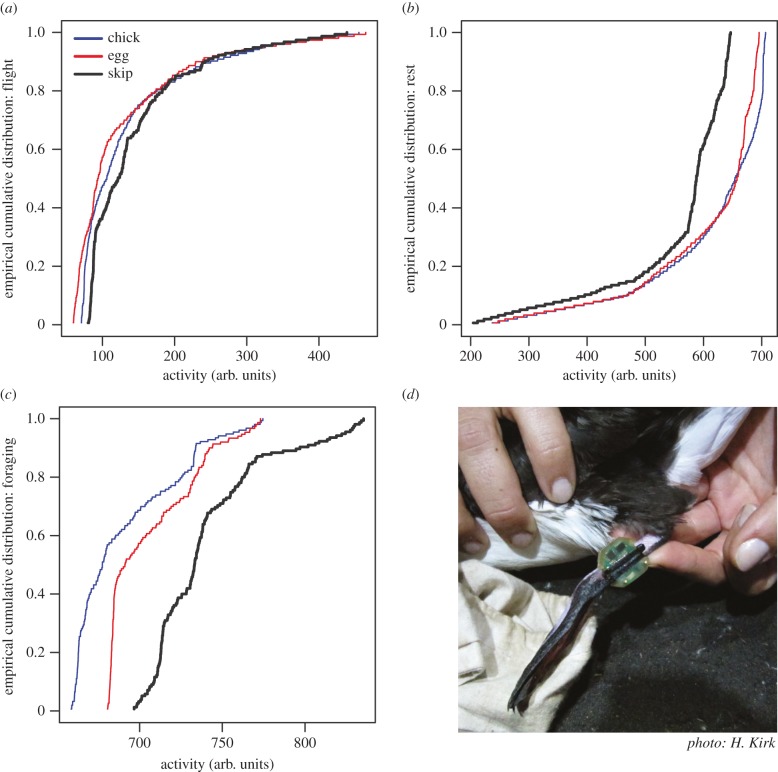
Activity-based segregation between three RP categories represented in blue (birds with a chick), red (egg failed) and black (skipped breeding). Empirical cumulative distributions of mean activity patterns are represented for (*a*) flying, (*b*) resting and (*c*) foraging times; (*d*) Manx shearwater with a geolocator on its leg.

To understand the factors that shape the timing of these prior events, we turned to individual SID. These showed some trends, among high-frequency variation (electronic supplementary material, figure S3), with no strong evidence for sex effects across behaviour classes (flying, *p* = 0.0281; resting, *p* = 0.1925; foraging, *p* = 0.0375; electronic supplementary material, figure S4). The time-series analyses did reveal activity-based segregation between breeders and non-breeders (electronic supplementary material, figure S5). This pattern was confirmed by extracting the corresponding empirical cumulative distribution by activity according to RP (figure 2). All comparisons proved statistically significant at the Bonferroni-corrected 1% level. Birds that skipped breeding in any given season (a minority) spent a previous winter marked by more flying (figure 2*a*), less resting (figure 2*b*) and much more foraging (figure 2*c*) than birds that attempted breeding. In contrast, those hatching a chick had had a winter essentially marked by low foraging activity (figure 2*c*).

### (c) Long-term reproductive tactics and carry-over effects

Exploring how these reproductive tactics might have evolutionary stability, MECMR modelling shows that birds that skipped breeding were less likely to fail or skip in the following year (electronic supplementary material, tables S3 and S4). For both sexes, the best-fitting model included state-dependent transitions, showing that current breeding states influence subsequent states (electronic supplementary material, tables S6 and S7). While in females, these rates were also different in different years, there was no evidence of individual heterogeneity in survival or transition rates for either sex (electronic supplementary material, table S5). Altogether, skipping birds had in the long run the highest chance of being successful breeders (0.79, 95% confidence interval [95 CI]: [0.36,0.96]) followed by failed birds (0.61, 95 CI: [0.33,0.83]), while successful birds had the lowest chance (0.57, 95 CI: [0.45,0.69]: electronic supplementary material, tables S8 and S9).

## 4. Discussion

We used data from year-round on-board activity loggers (5 years, more than 100 individuals) to monitor remotely at-sea behaviour patterns and the timing of individual breeding and migratory events, in a trans-global migratory seabird, the Manx shearwater. We uncovered chained relationships between consecutive seasons, with compelling evidence for links between previous breeding phenology, winter at-sea behaviour, and subsequent breeding performance. This confirms that some intra-specific variations in RP, which have not been fully explained by conventional theories [19], may be due to COEs.

First, timing differences in breeding one year are also related to RP during the next, with earlier egg-laying, chick-hatching and fledging all predicting greater RP in the following season because early birds are generally better [20]—perhaps owing to differences in age, experience or underlying individual quality [21]. Second, despite generally high breeding success, and hence unbalanced data with few individuals failing, birds that skip breeding show predictably higher activity (most probably higher foraging effort) in the previous winter (see also [11]). This suggests that individuals try to compensate either for their own poor condition (itself potentially an effect of previous breeding), or for poor foraging conditions encountered in winter. In either case, whatever causes increased over-winter activity carries over into an effect on breeding success during the following season. Third, we find that shearwaters occasionally skip breeding and that when they do, they go on to have better RP next year. This shows that release from the cost of breeding in one season can carry over into enhanced future success. Thus, while skipping may be an adaptive response to being in poor condition, it may also reflect the resort of relatively poor-quality individuals (in other long-lived seabirds individuals may differ consistently in their average skipping propensity and in their response to environmental variation: e.g. [22]).

Our MECMR models further provide strong support for the proposition not only that current breeding state affects subsequent breeding states, but also that the amount of current reproduction negatively affects subsequent breeding states: a COE reflecting the trade-off between current and future reproduction as predicted by life-history theory [23].

## Supplementary Material

Supplementary Information

## Data accessibility

Data are available in the electronic supplementary material.
