# Supplementary material for: Breeding phenology and winter activity predict subsequent breeding success in a trans-global migratory seabird
Source: Biol Lett. 2015 Oct;11(10):20150671. doi: 10.1098/rsbl.2015.0671 (PMC4650180; doi:10.1098/rsbl.2015.0671)
Supplement: Supplementary Information [file rsbl20150671supp1.pdf]

Determination of at-sea behaviour

At-sea behaviour was measured by individual salt-water immersion data recorded by the geolocators. These data range from 0 (= complete dry) to 200 (= complete wet) 3 sec sample moments within each 10 min bin (thus, data resolution is 10 min). To classify behaviour into flying, resting and foraging, we used the previously available method (reported in [1, 2]). Briefly, complete dryness is considered to be "FLIGHT", complete wetness is "REST" (on surface water) and intermediate values from 1-199 is "BEING ACTIVE = FORAGING ACTIVITY". This method has been shown to correlate with landing and take off patterns associated with foraging, since any intermediate value will (normally) indicate sea-air transitions which are known to be of prime importance to foraging costs in other procellariiformes [1].

Determination of reproductive success

Three possible outcomes of reproduction were here tallied according to what was found in the nest: (i) the egg hatched and the chick fledged, or was verified as healthy at the last inspection late-on in rearing and so almost certainly fledged (our "CHICK" category); (ii) an egg was laid but then failed ("EGG"); or (iii) no egg was found ("SKIP"). All nests, but six, were monitored until fledging. These six exceptions were monitored until a few days before expected fledging, a time late on in rearing when breeding failure is extremely rare away from puffinosis areas (as was our study site), and all six chicks were already of sufficient size to fledge at this last inspection. These six chicks almost certainly fledged, and were included in category "CHICK".

Note that nest predation on this colony is extremely rare – there are no rats or other terrestrial predators. Occasionally gulls and jackdaws can be seen dragging chicks out of burrows but this is very rare – and this does not happen at egg stage (it requires the chick to walk towards the entrance of the burrow). However, eggs can be broken if another adult shearwater enters the nest and starts digging when the egg is being neglected. However, this is easy to detect as the egg goes missing or is broken. In our study, failed eggs were either eggs that were incubated but never hatched, or eggs that went missing or

were broken. However, all these events are associated with parents failing to incubate the egg properly without gaps between incubation shifts – leading to the egg not hatching, or to the egg being left alone in the burrow and therefore being damaged by other birds. Therefore this has to do with incubation quality and could be related to prior activity (e.g. parents could be in a poor body condition, requiring them to cut short their incubation stints to feed); it should not be adding any random noise to our results.

To minimise disturbance, we made sure that several steps were taken during our daily visits: 1) we used 'knock-down tags' to detect changes in occupancy [3, 4]; 2) we used 'short-access tunnels', allowing us to have rapid access to chicks or adults, reducing over all disturbance [5, 6]; and 3) many of our study birds already carry a radio frequency identification tag [7], allowing us to check bird identity without handling. Knock-down sticks allow us to reduce disturbance because we only need to inspect nests when the tags were disturbed at the entrance showing that an adult had entered or exited the burrow (these can be disturbed by prospectors, but this does not happen often). Short-access tunnels are a widely used method (e.g. [8]) to reduce disturbance as described by [5]. This system facilitates easy access to burrow chambers as we make a hole above the chamber and put a lid on which is covered by vegetation, hiding the holes completely [8]. The holes were made during daytime when adults are absent and had no negative effects on burrow occupancy [8]. The goal of our daily inspections was to identify 'egg neglect' and the breeding progress (egg lay date, start of incubation, hatch day, fledge day). Thus, we did not have to remove birds from their burrow during daily inspections, but only to confirm whether or not an adult was in the burrow.

Data analyses

To assess predictive power of phenology on individual RP, we employed a supervised machine-learning algorithm based on adaptive boosting [9]. The classifier was trained on a set of features to predict individual RP as one of these three categories: "CHICK", "EGG", or "SKIP". Nine features were included: we considered both prior- and post-breeding phenology (laying, hatching and fledging dates), as well as migratory phenology as extracted above (dates of colony departure, arrival at wintering grounds, departure

from wintering grounds and colony arrival). The SAMME algorithm [10] was used as prediction is on > 2 (3 here) categories. The classifier's accuracy was determined by 10-fold cross-validation, where the algorithm is trained on nine tenths of the data and the last decile is used to compute a confusion matrix, the procedure being repeated for all ten subsets. This process was repeated 1,000 times to assess classification accuracy.

To understand how wintering behaviour affects RP, we analysed behavioural patterns based on saltwater-immersion data. Because these data show high-frequency variability, a de-noising procedure was first used to extract nonlinear trends without any reference to breakpoints identified above. To this effect, a time-series additive decomposition was performed to extract nonlinear trends for each track. Cumulative distributions of de-noised data were then extracted and averaged for "CHICK", "EGG" or "SKIP". The Kolmogorov-Smirnoff test was used to assess significance.

Year-to-year reproductive data can also be summarised as a transition matrix giving the frequencies of RP state changes from one year to the next. To this effect, sample size was expanded to include an additional 88 individuals (47 males, 41 females) whose breeding progress and breeding performance were monitored, but which had not been tracked with Geolocators. Multi-event capture-mark-recapture (MECMR) models [11] were used to estimate transitions rates among "SKIP", "CHICK", "EGG" and "DEAD" (= not recaptured). Survival rates were either constant or varying as a function of state and/or time; the best transition rate model was identified using Akaike Information Criterion with small sample correction.

A note on the breeding habits of Manx shearwaters

Unlike some albatrosses, Manx shearwaters do normally breed every year. In fact, their subsequent breeding success is dependent on the amount of investment that breeders made in the previous year. If Manx shearwaters use the "by-product breeding pattern", we should expect to see the simpler transition between SKIP/CHICK and CHICK/SKIP once they reach maturity, which we did not observe here. Thus, we suggest that Manx shearwaters do try to breed every year, and when their previous breeding event was costly, they try to regain body condition during winter as much as possible, but do not

manage to regain their condition by the next spring and therefore decide to skip (or could not manage) breeding instead.

A note on the device effects

We have used a measure of 'breeding success' of individuals that carried a geolocator in our experimental colony (North Haven) to assess the overall impact of our tracking work by comparing it with a neighbouring unmanipulated plot (Isthmus) which has been monitored for many years by the National Nature Reserve staff (Bu□che et al. 2013 in JNCC Contract Report: available from 1995 to 2013) to provide estimates of shearwater productivity on the island. Doing this allows a large sample of breeding attempts to be compared, but it restricts us to using the same measure of breeding success as in those studies. This measure was the number of chicks raised per egg laid, and is therefore not quite the same as that used for the carry-over analyses at the core of our study (which includes birds that skip). Thus, for the impact assessment comparison only we used the same measure as the island staff – chicks raised per egg laid – which across the duration of our study was 0.86, compared to 0.63 in the unmanipulated plot for the same period (2009-2013).

We did not detect any measurable effect in breeding performance (number of chicks per laid egg) in this study (Table S1) as agreed with another geolocator study in Manx shearwaters [12]. However, negative effects of equipping year-round geolocator in hormonal responses have been reported [13]. Thus some caution in interpreting the results may be suggested.

Comparing breeding success at unmanipulated and manipulated plots

Breeding success of Manx shearwaters from 2009 to 2014 in unmanipulated plot (Isthmus: data are available until 2013: [14]) and in experimental plot (North Haven: individuals used in this study).

Plot	Year	No. Laid	No. Fledged	% Eggs (laid) Fledged
Isthmus (unmanipulated)	2009	114	67	0.59
	2010	115	70	0.70
	2011	99	61	0.69
	2012	91	37	0.55
	2013	87	52	0.60
	2014	NA	NA	NA
			Mean	0.63
North Haven (geolocator carried)	2009	13	12	0.92
	2010	22	19	0.86
	2011	11	10	0.91
	2012	24	24	1.00
	2013	38	32	0.84
	2014	32	20	0.63
			Mean	0.86

Table S1. Number of individuals tracked. A total of 108 birds were tracked between 2009 and 2014; three of them were tracked for > 1 season, which is why 111 bird-seasons were observed.

Migration year	Number of individuals	CHICK	EGG	SKIP	Male	Female	Sex unknown
2009-2010	14	7	5	2	2	2	10
2010-2011	23	17	2	4	7	4	12
2011-2012	12	10	1	1	4	8	0
2012-2013	24	10	9	5	11	13	0
2013-2014	38	20	12	6	14	13	11

Table S2. Confusion matrix demonstrating the performance of the adaptive boosting algorithm. Results are based on a ten-fold cross-validation experiment. CHICK indicates individuals that successfully had a chick, EGG indicates individuals that failed breeding during incubation and SKIP indicates individuals that skipped breeding.

		Observed		
		CHICK	EGG	SKIP
Predicted	CHICK	56	15	16
	EGG	6	13	2
	SKIP	2	1	0

Table S3. Observed transition matrix in RP on the extended sample for (a) female and (b) male.

(a) Female

		Year $y + 1$ →			
		CHICK	EGG	SKIP	DEAD
Year y →	CHICK	39	14	8	18
	EGG	8	1	2	3
	SKIP	3	0	0	3
	DEAD	-	-	-	-

(b) Male

		Year $y + 1$ →			
		CHICK	EGG	SKIP	DEAD
Year y →	CHICK	39	16	6	19
	EGG	8	1	1	6
	SKIP	3	1	0	3
	DEAD	-	-	-	-

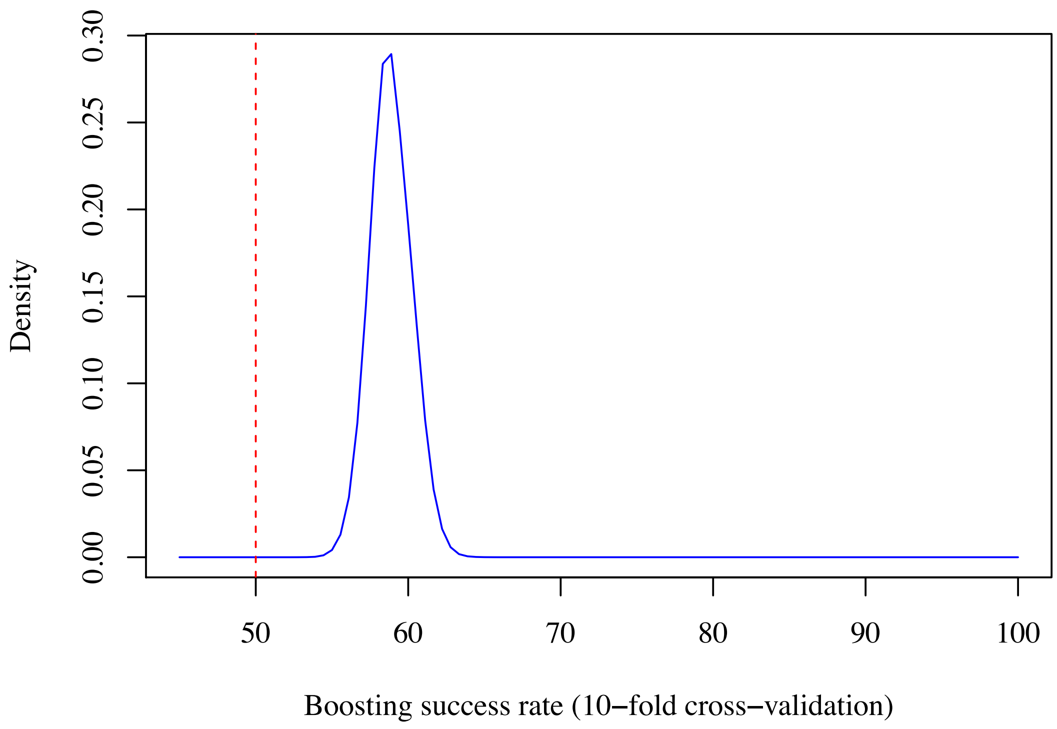

Figure S1. Sampling distribution of the success rate of the SAMME classifier assessed by ten-fold cross-validation. This distribution was obtained by running the cross-validation 1,000 times.

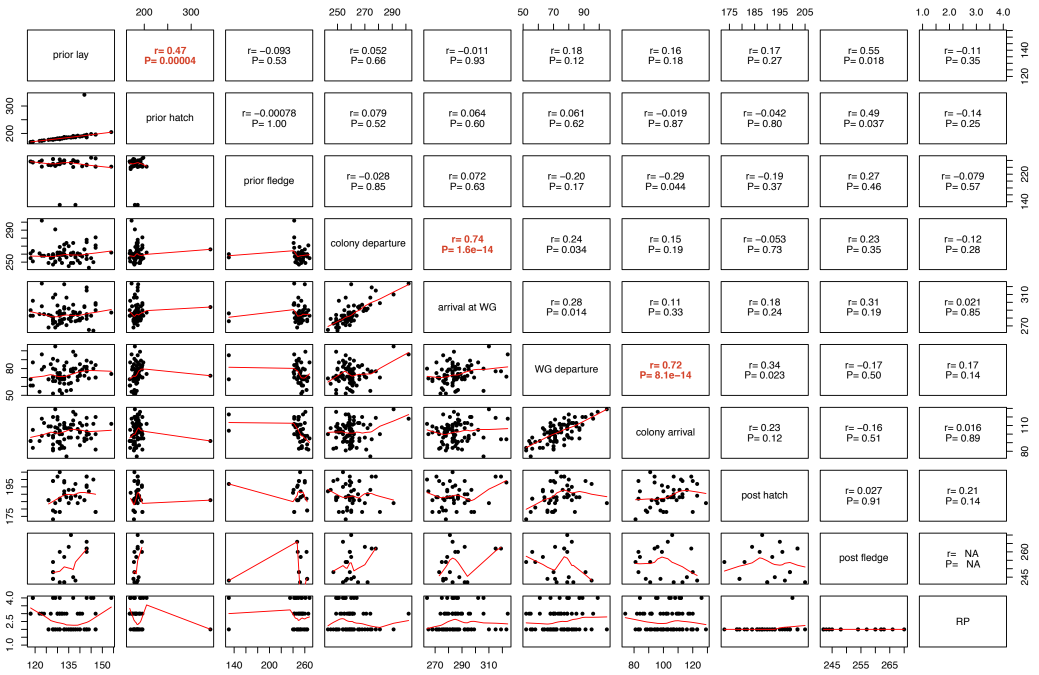

Figure S2. Correlation among the features used to train the classifier. Feature names are on the diagonal of the correlation matrix; correlations and their *P*-values are shown in the upper triangular matrix (in red if significant at the 1% level), while the corresponding data are shown in the lower triangular matrix. Dates are represented as day number in each year (*e.g.*, the 31st of December is day 365). RP is reproductive performance.

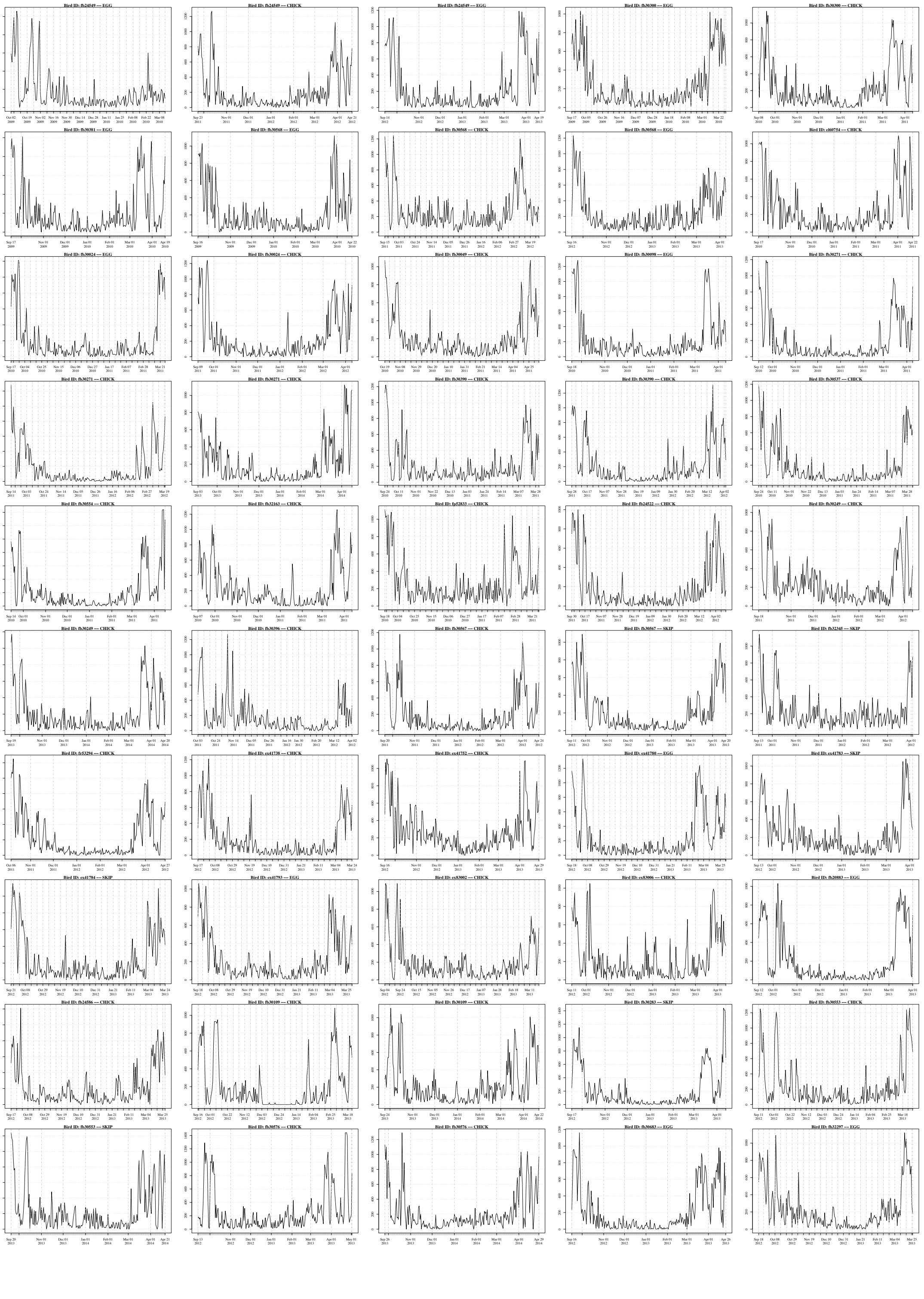

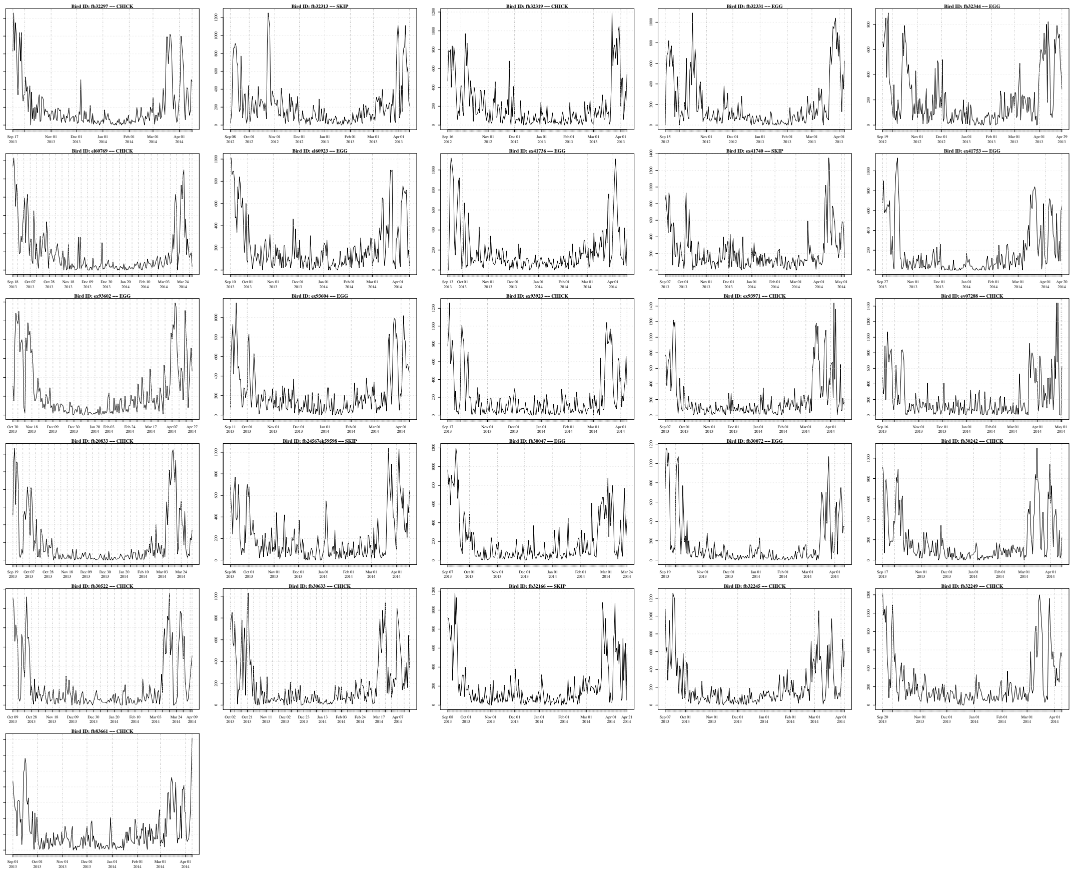

Figure S3a. Flight patterns extracted from saltwater-immersion data for each bird. Level of activity is represented as a function of time. Individual RP is indicated.

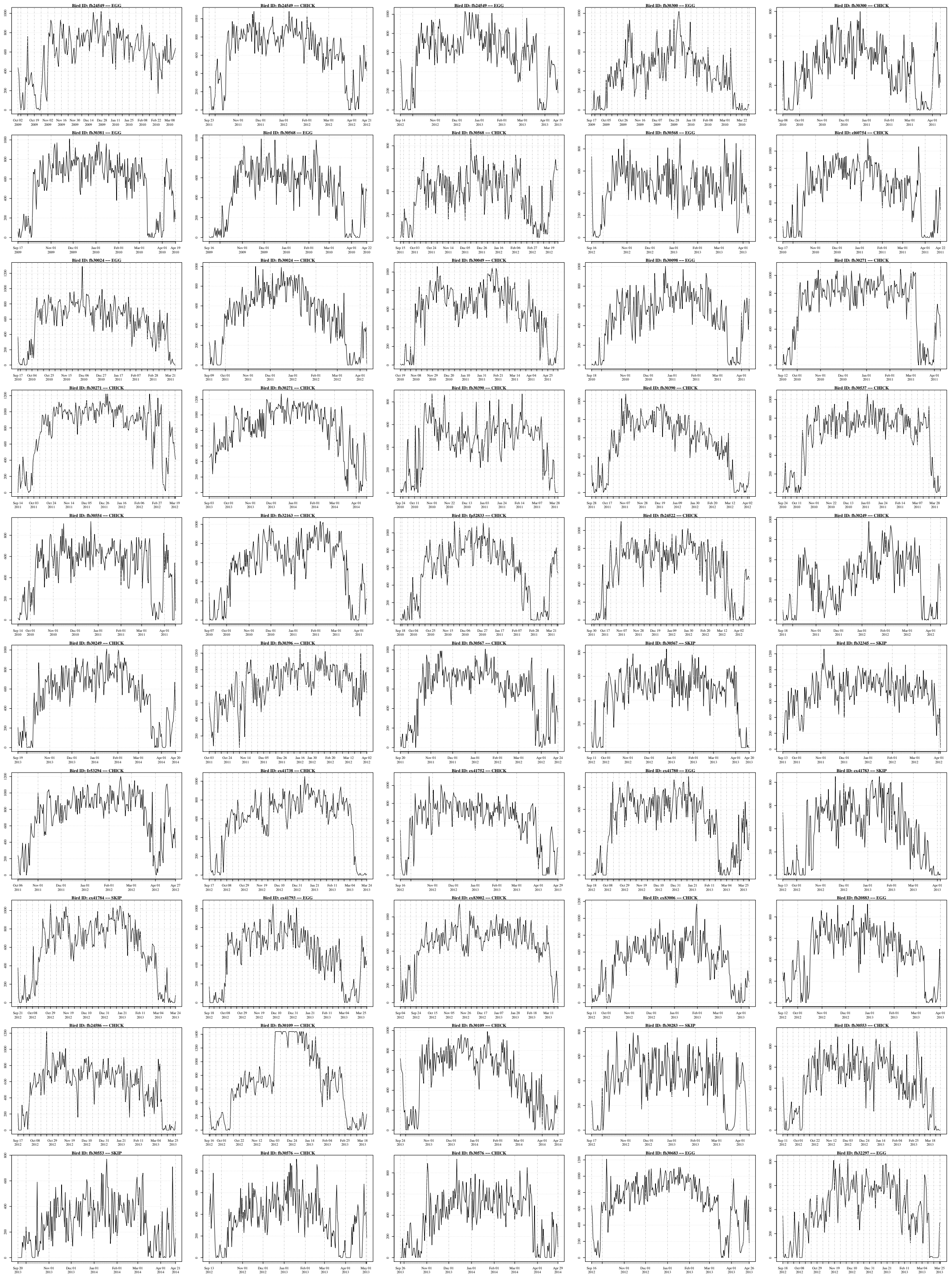

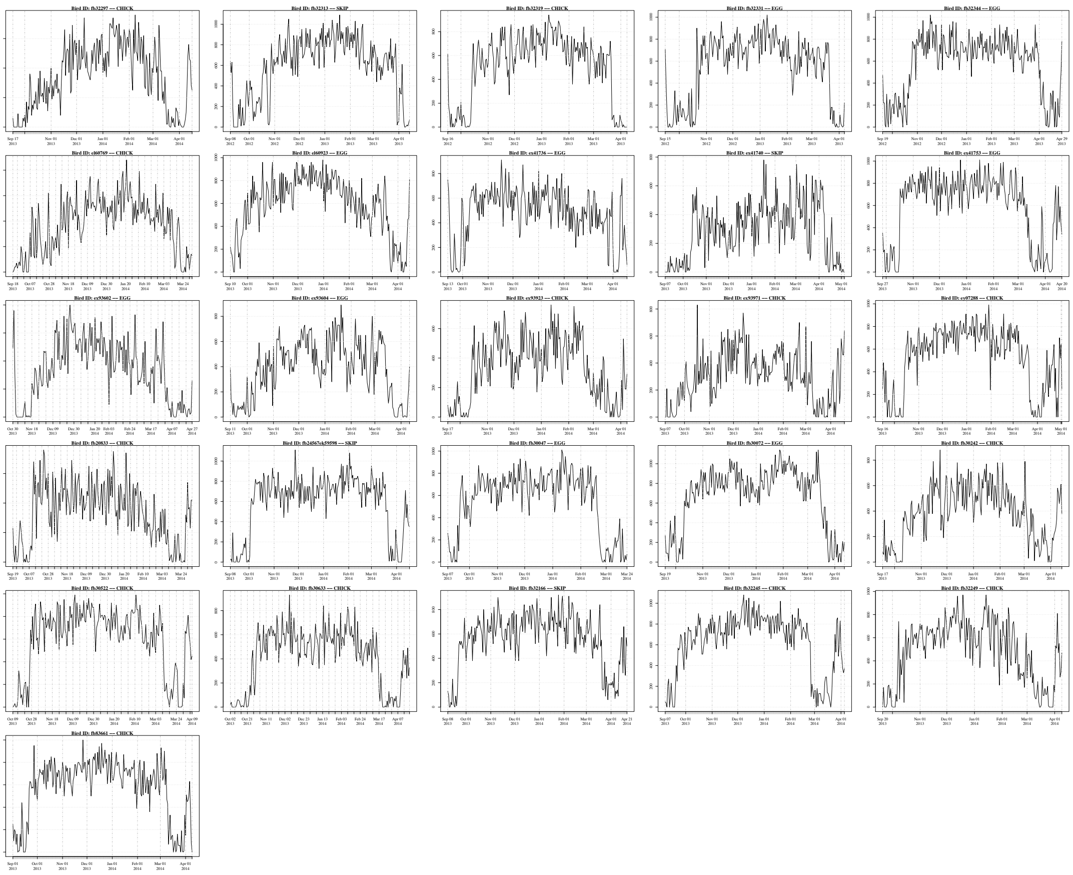

Figure S3b. Resting patterns extracted from saltwater-immersion data for each bird. Level of activity is represented as a function of time. Individual RP is indicated.

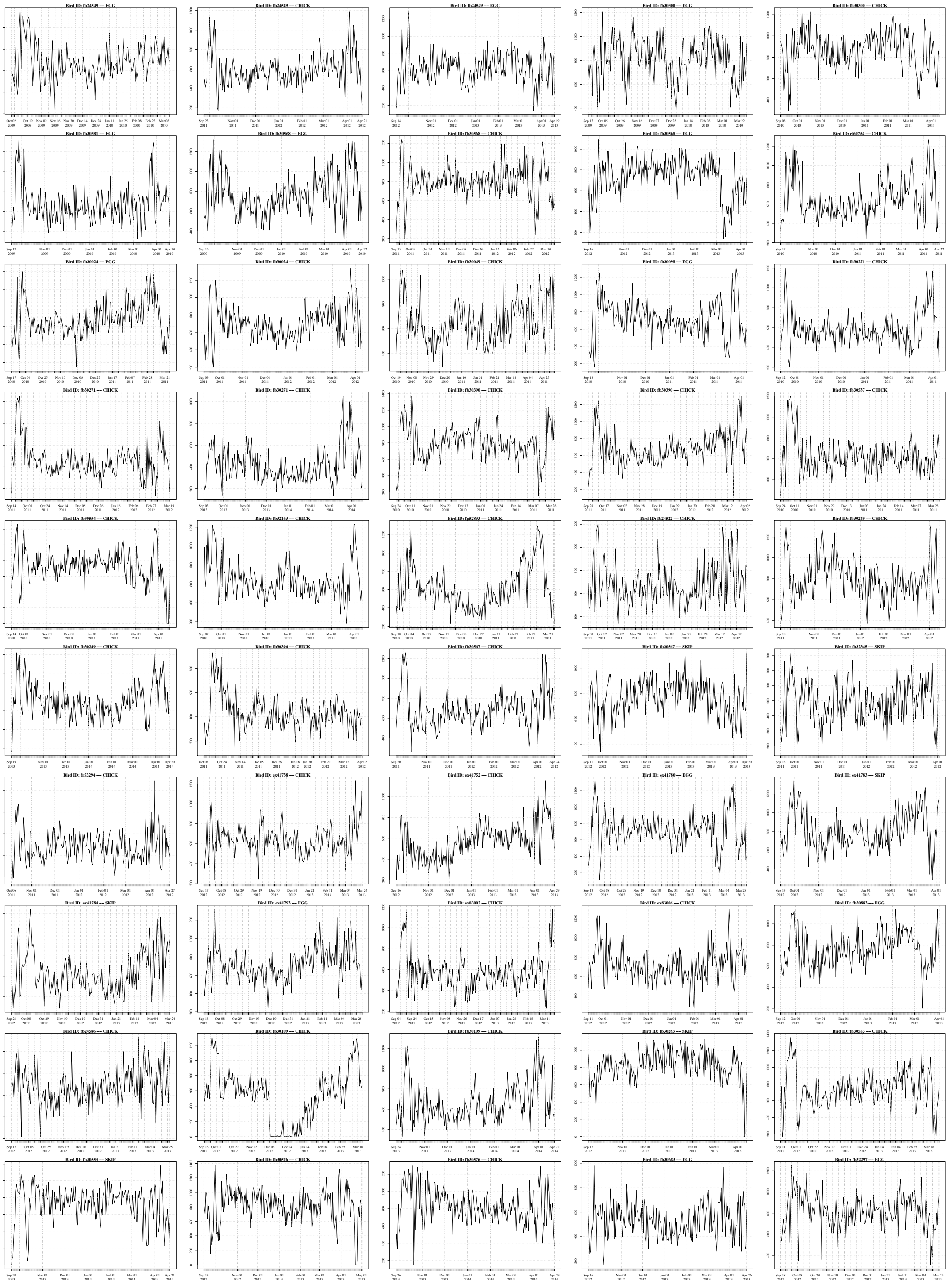

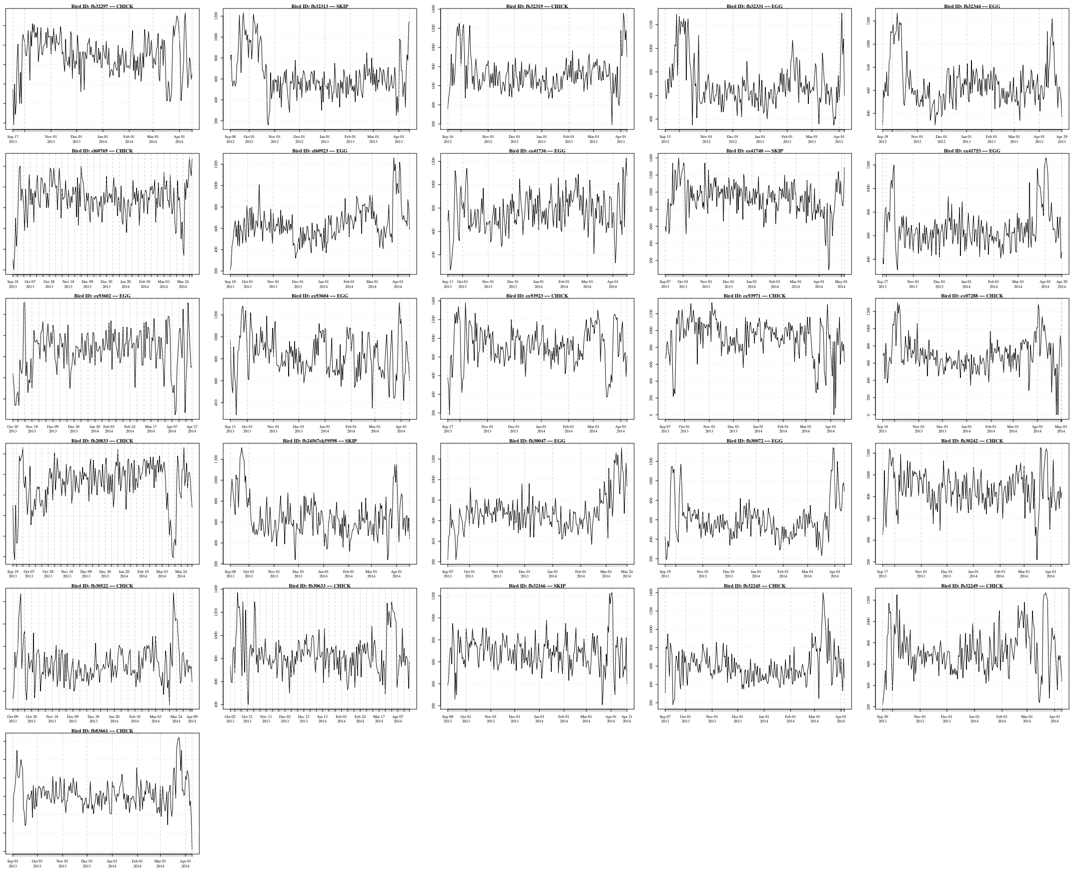

Figure S3c. Foraging patterns extracted from saltwater-immersion data for each bird. Level of activity is represented as a function of time. Individual RP is indicated.

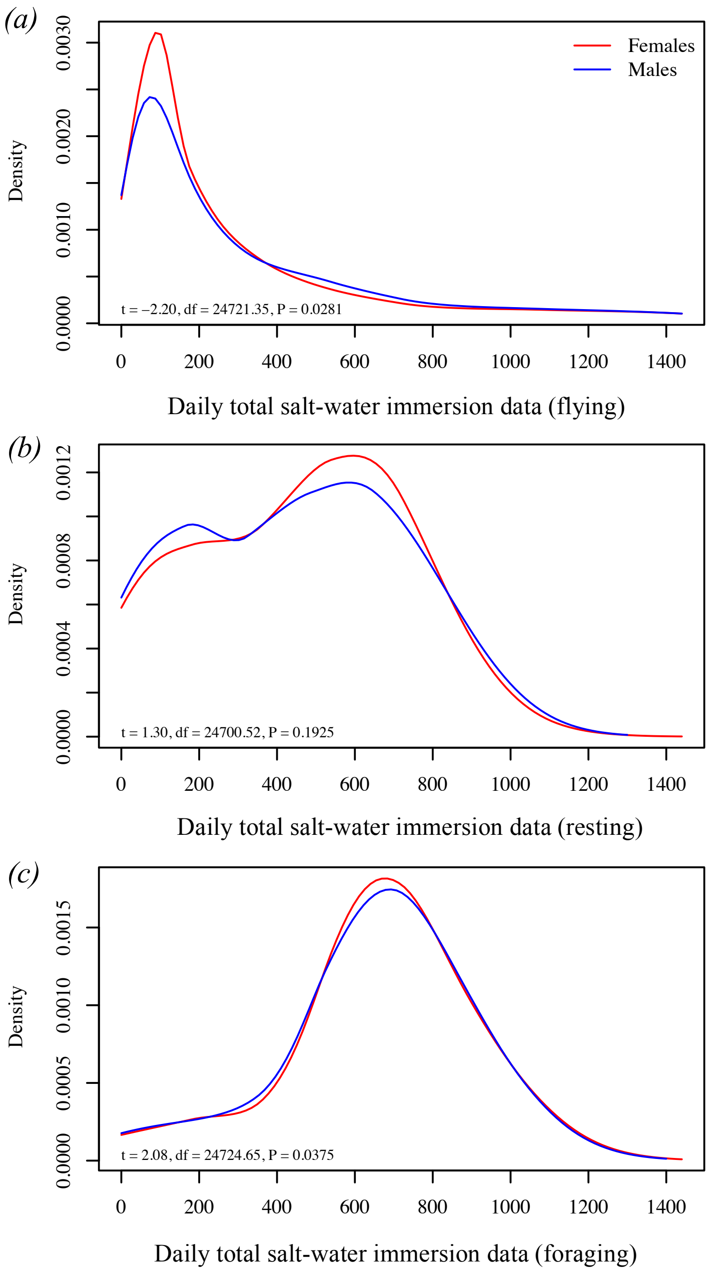

Figure S4. Daily distributions of salt immersion data (SID) between the sexes by activity type: (*a*) during flying, (*b*) during resting and (*c*) during foraging. Tests results are shown as insets in the bottom left of each panel. The *x*-axis represents the number of times that each type of activity (flying: SID = 0, resting: SID = 200, foraging: SID in]0,200[) is recorded per day per bird.

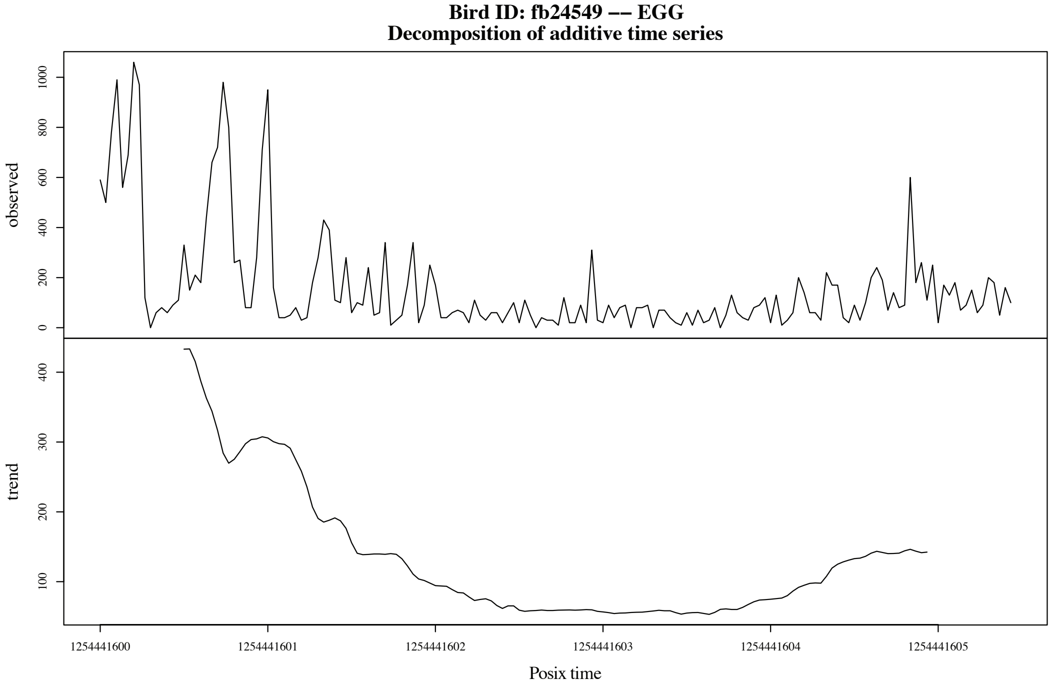

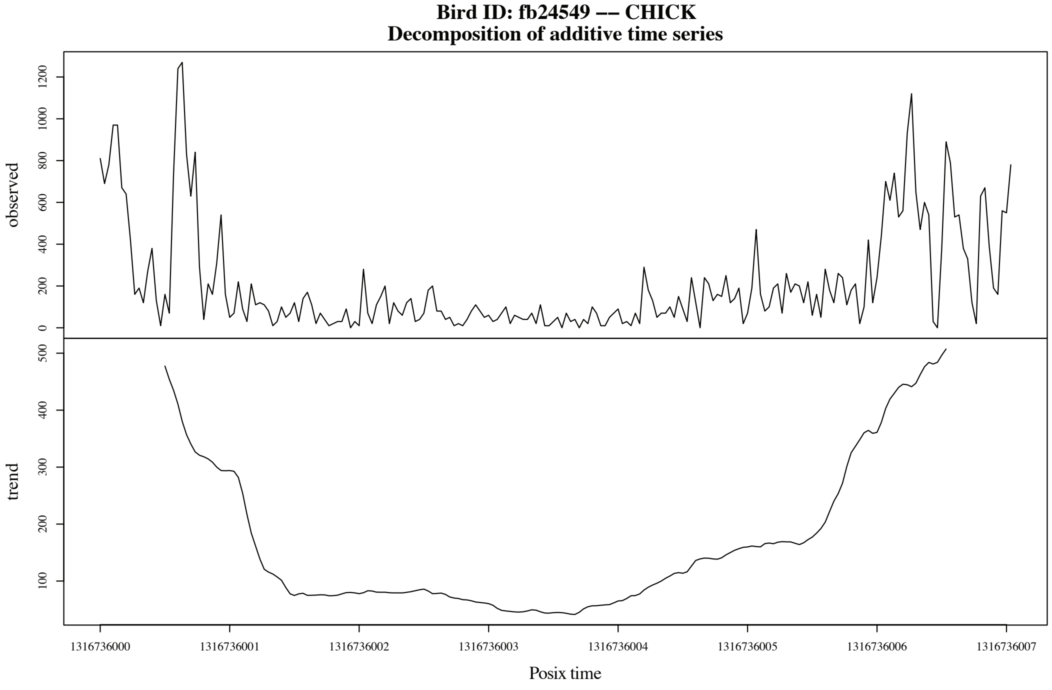

Figure S5. Two examples of data de-noising by nonlinear trend extraction with additive time series decompositions.

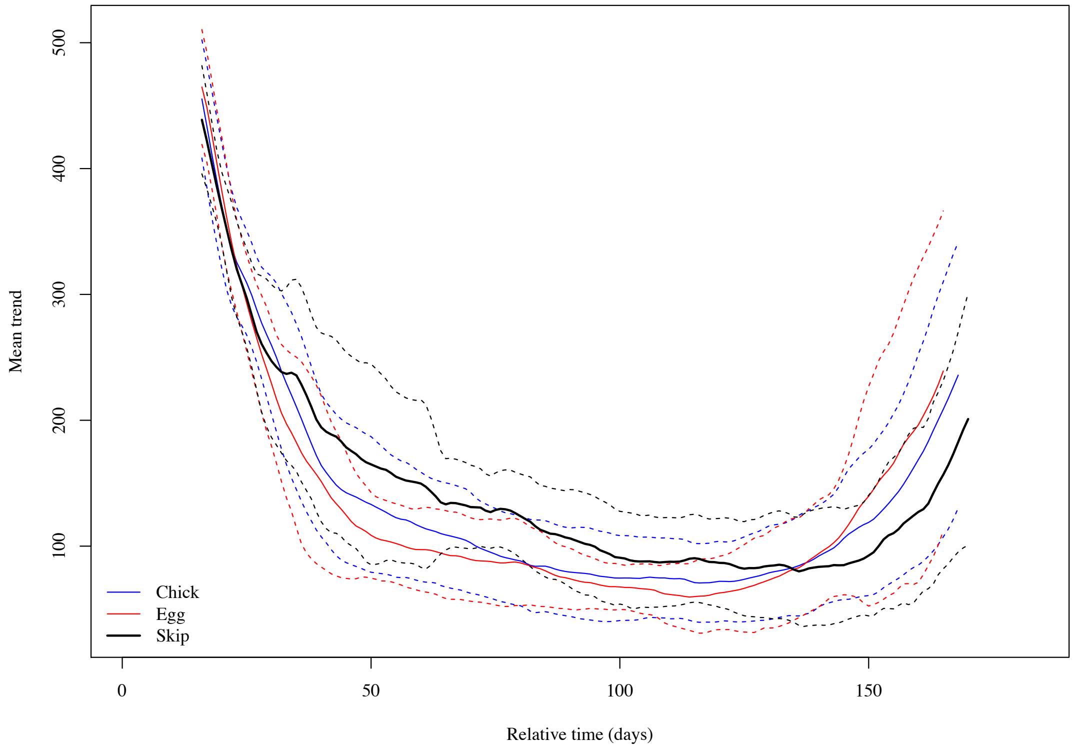

Figure S6a. Mean flying patterns obtained for birds with a chick (blue), egg failed (red) and for skipped birds (black). Mean values were obtained from activity plots presented in Fig 3a. Broken lines represent mean value ±1 SD.

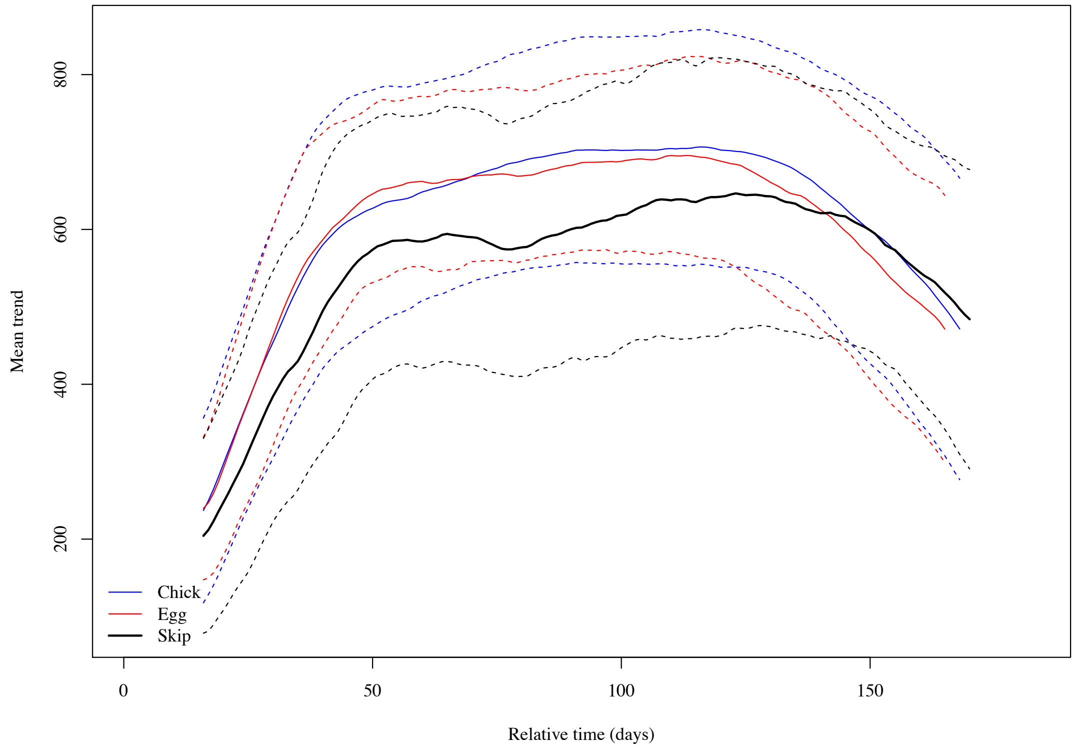

Figure S6b. Mean resting patterns obtained for birds with a chick (blue), egg failed (red) and for skipped birds (black). Mean values were obtained from activity plots presented in Fig 3b. Broken lines represent mean value ±1 SD.

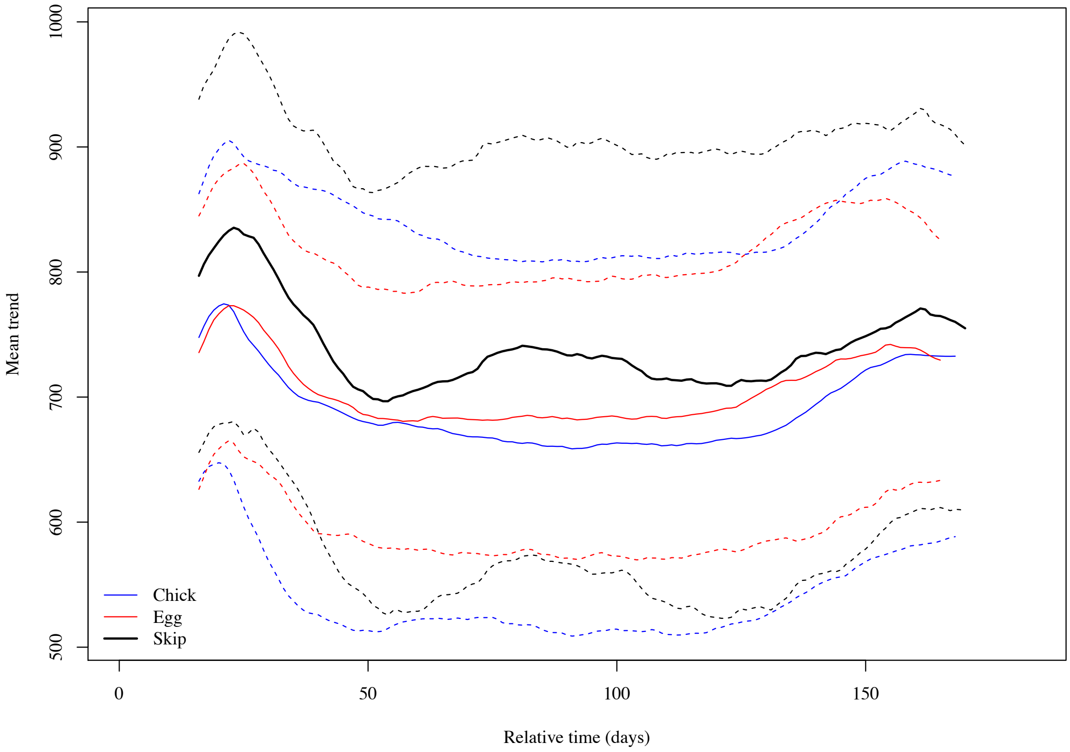

Figure S6c. Mean foraging patterns obtained for birds with a chick (blue), egg failed (red) and for skipped birds (black). Mean values were obtained from activity plots presented in Fig 3c. Broken lines represent mean value ±1 SD.

Multi-event model framework for estimating state transition rates

Multi-event capture-mark-recapture (MECMR) is a modelling framework widely used to estimate state-dependent demographic rates of interest (*e.g.* survival) together with transition rates between different "states" individuals occupy (*e.g.* being infected or not), while explicitly accounting for imperfect (less than 1) and heterogeneous (biased) detectability of marked individuals, and uncertainty in the assignment of the state to an individual. Given their breeding status (*i.e.* skipped breeder, breeder with an egg, breeder with a chick), birds can be assigned to different states, and multi-event models provide an ideal framework for estimating transition rates (from state to state) and survival probabilities simultaneously in the same model. Furthermore, as different constraints can be imposed on state-dependent survival and transition parameters, multi-state models provide a rigorous method of evaluating the fitness consequences of transition rates.

In MECMR models, at each capture occasion an individual can occupy one amongst a finite set of mutually exclusive states. Between subsequent capture occasions, individuals move independently between these states [11]. However, a state is not always possible to assign when an individual is captured. Thus, at each capture occasion, we observe an event rather than a state. Events are related to the true, but not necessarily known, state of the individual through a series of conditional probabilities [15, 16]. The MECMR model we develop here uses four exclusive states that an individual can occupy at each capture occasion (which is the breeding season): (1) being breeder with a chick (state "CHICK"), (2) being a failed breeder, producing only an egg (state "EGG"), (3) skipped breeder (state "SKIP"), (4) dead (state "DEAD").

An individual can occupy only one state in a given breeding season. Transitions among these four states (*i.e.* "CHICK", "EGG", "SKIP, "DEAD") happen between two subsequent breeding seasons, with the state 'DEAD' being an absorbing state (a dead individual cannot move to another state). Transitions are modelled as a two-step process composed of the probability of survival over the annual time interval, followed by the probability of transitioning among live states. The recapture of the marked individuals is described in the event matrix, where an individual that is alive (*i.e.* occupying alive state) can be either captured, or not captured. There are four possible events, related to one or more real underlying states:

0 = individual is not captured ("DEAD", "CHICK", "EGG", "SKIPPED")
1 = individual is captured at the nest, and it produced an egg in that breeding season ("EGG")
2 = individual is captured at the nest, and it produced a chick in that breeding season ("CHICK")
3 = individual is captured at the nest, but without an egg or chick ("SKIPPED")

We coded the capture histories of males and females using the four event codes shown above (0, 1, 2, 3). We treated females and males in two separate analyses to avoid any problems related to non-independence between males and females (*i.e.* they may belong to the same breeding pair).

Specification of parameters and the model structure

Following notation in Pradel (reference [3]) our model is defined with three types of parameters: (1) initial state probabilities, represented in a vector of probabilities, (2) transition probabilities involving: survival probabilities (ϕ), and between-state transition probabilities (ψ); and (3) recapture probabilities (p).

(ϕ) Survival probabilities

	SKIP	EGG	CHICK	DEAD
SKIP	y	-	-	1 - y
EGG	-	y	-	1 - y
CHICK	-	-	y	1 - y
DEAD	-	-	-	1

(ψ) Transition

	SKIP	EGG	CHICK	DEAD
SKIP	1 - y	y	y	-
EGG	1 - y	y	y	-
CHICK	1 - y	y	y	-
DEAD	-	-	-	1

(p) Recapture probabilities

	0	1	2	3
SKIP	1 - y	-	-	b
EGG	1 - y	b	-	-
CHICK	1 - y	-	b	-
DEAD	1 - y	-	-	-

Model covariates and model selection process

There is no specific goodness of fit (GOF) test for MECMR models. Thus, we assessed the fit of the general mark-recapture assumptions to our data by assessing the GOF of the single state Cormack-Jolly-Seber (CJS) model [17]. The CJS model assumes all animals present at the same sample occasion have equal future survival and recapture probabilities regardless of past history and capture in the current sampling occasion. These assumptions were tested using program U-SURGE [18]. None of the components of the test returned significant results.

We considered four possibilities for the variation of each parameter (recapture, survival, transition): state (*i.e*. parameter varies according to a state individuals is), time (*i.e*. parameter varies in time), time + state, constant (parameter is the same for all individuals, and constant in time). We used a 3-stage model selection process [19]: first we modelled recapture rates as constant, or as varying in relation to state, or time (yearly variation), while keeping survival and transition rates fully parameterised (state + time). Next, we used the best recapture rate model identified in the first state (*i.e.* the model with the lowest Akaike Information Criterion for small sample sizes ($QAIC_c$) correcting for overdispersion by including an estimate of model deviance (c-hat = model deviance/*df*) for the global model), to model survival rates as constant, or as varying in relation to state and time. Finally, with recapture and survival rates parameterised according to the best models identified above, we modelled transition rates as constant, or varying in relation to time and departure state (the state an individual transitions from). In total, we performed model selection on a candidate list of four different recapture, four different survival and four different transition rate models (Table S4). Model selection was based on QAICc [20]. Normalised QAICc weights (w_i) were used as a measure of relative support for each model.

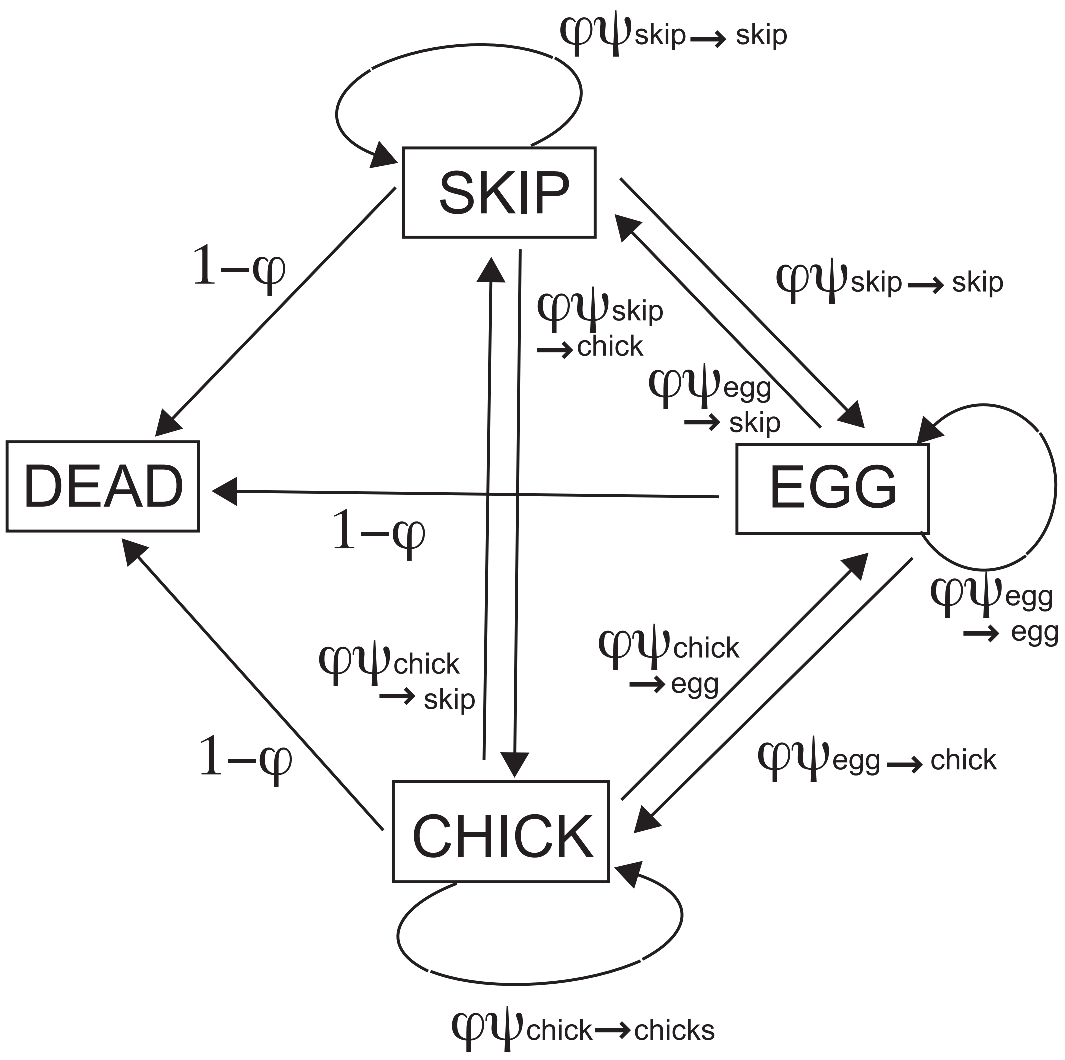

Figure S7. A diagram of the between-state transition process and the structure of the observation process used to estimate transition rates. RP (*t*): reproductive performance in year *t*, RP (*t*+1): reproductive performance in year *t* + 1. Chick: individuals that have a chick in a given year, Egg: individuals that failed breeding in a given year, Skip: individual that skipped breeding but stayed in a burrow in a given year. Dead: individuals that were not captured in a given year. ϕ: survival rate; Ψ: transition rate.

Table S4. Model notation describing the recapture, survival and transition rate models included in the candidate list for multi-event modelling of survival and transition rates in Manx shearwaters.

Recapture	Survival	Between-state transition
state	Best model of recapture	Best model of recapture
t	state	Best model of survival
state + *t*	*t*	state
constant	state + *t*	*t*
	constant	state + *t*
		constant

Class mixture model

To include the possibility of intrinsic individual differences that can potentially influence survival and breeding-state transition probabilities, we implemented mixture models that incorporated two distinctive quality classes of individuals. We call these higher (H) and lower (L) quality individuals. We did not have any prior information on which class individuals belonged to, so we treated these classes as hidden states. Thus, we extended the main model outlined above to include 1 dead state, and 6 alive states: skipped breeder in class H; skipped breeder in class L; breeder with an egg in class H; breeder with an egg in class L; breeder with a chick in class H; breeder with a chick in class L. Survival and transition between breeding states were allowed to happen only within the same heterogeneity class (*i.e.* a bird stays in the same class and can change breeding state within that class, or die). Observations (events) are the same as in the previous modelling framework.

To test if there is evidence for heterogeneity in survival or transition rates, we imposed the parameters of the relevant rate to be different between the two classes, or to be same for all individuals (*i.e.* there is no heterogeneity). In both sexes, we kept the main model structure (*i.e.* variation in parameters) as selected in the previous model selection,

without heterogeneity, and then allowed the initial state, survival and/or transition rates to vary according to class of heterogeneity. Model selection was, as above, based on normalised QAICc weights (w_i).

Table S5. Summary results of the multi-event mark-recapture analysis with and without heterogeneity to estimate recapture, survival and transition rates between a given year and a following year in Manx shearwaters.

(a) Females

Model	Model Structure							
	p	Φ	ψ	*K*	dev	QAICc	Δ*i*	w_i
1	**No heterogeneity**	**No heterogeneity**	**No heterogeneity + state dependent**	**10**	**291.21**	**313.19**	**0.00**	**0.91**
2	No heterogeneity	Heterogeneity	No heterogeneity + state dependent	12	291.21	318.07	4.88	0.08
3	Heterogeneity	No heterogeneity	Heterogeniety + state dependent	17	282.40	322.28	9.09	0.01
4	Heterogeneity	Heterogeneity	Heterogeniety + state dependent	19	282.85	325.50	12.30	0.00

(b) Males

Mode l	Model Structure							
	p	Φ	ψ	*K*	dev	QAICc	Δ*i*	w_i
1	**No heterogeneity**	**No heterogeneity**	**No heterogeneity + state dependent**	**10**	**291.21**	**317.48**	**0.00**	**0.91**
2	No heterogeneity	Heterogeneity	No heterogeneity + state dependent	12	291.21	322.34	**4.86**	**0.08**
3	Heterogeneity	No heterogeneity	Heterogeniety + state dependent	17	282.40	328.30	**10.82**	**0.00**
4	Heterogeneity	Heterogeneity	Heterogeniety + state dependent	19	282.85	333.00	**15.52**	**0.00**

See Figure S6 for model notation. *K*: number of estimable parameters, dev: deviance, QAICc: Akaike's information criterion for small sample sizes ($QAIC_c$) correcting for overdispersion by including an estimate of model deviance (c-hat = model deviance/*df*) for the global model, Δ*i*: the QAICc difference between the current model and the model with the lowest QAICc value, w_i: Akaike weight, state: state dependent rates, constant: constant rates, *t*: time-dependent rates.

Table S6. Summary results of the multi-event mark-recapture analysis to estimate recapture, survival and transition rates between a given year and a following year in female Manx shearwaters.

	Model Structure							
Parameter	p	Φ	ψ	*K*	dev	QAICc	Δ*i*	w_i
Recapture rate (p)	**state**			**22**	**272.01**	**326.23**	**0**	**0.74**
	t			20	281.51	329.83	3.6	0.12
	state + *t*			24	269.64	330.01	3.79	0.11
	constant			26	265.78	332.56	6.33	0.03
Survival rate (Φ)	**state**	**constant**		**16**	**277.47**	**314.6532**	**0.00**	**0.81**
	state	state		18	275.88	318.5182	3.86	0.12
	constant	constant		14	288.90	320.8234	6.17	0.04
	state	*t*		20	273.39	321.707	7.05	0.02
	constant	*t*		18	282.06	324.697	10.04	0.01
	constant	state		16	287.93	325.1146	10.46	0.00
	state	state + *t*		22	272.01	326.2285	11.58	0.00
	constant	state + *t*		20	281.51	329.826	15.17	0.00
Transition rate (ψ)	**state**	**constant**	**state + *t***	**16**	**277.47**	**314.6532**	**0.00**	**0.52**
	state	constant	state	12	289.27	316.1347	1.48	0.25
	state	constant	constant	6	303.58	316.3103	1.66	0.23
	state	constant	*t*	11	302.40	326.8032	12.15	0.00

See Figure S6 for model notation. *K*: number of estimable parameters, dev: deviance, QAICc: Akaike's information criterion for small sample sizes ($QAIC_c$) correcting for overdispersion by including an estimate of model deviance (c-hat = model deviance/*df*) for the global model, Δ*i*: the QAICc difference between the current model and the model with the lowest QAICc value, w_i: Akaike weight, state: state dependent rates, constant: constant rates, *t*: time-dependent rates.

Table S7. Summary results of the multi-event mark-recapture analysis to estimate recapture, survival and transition rates between a given year and a following year in male Manx shearwaters.

	Model Structure							
Parameter	p	Φ	ψ	*K*	dev	QAICc	Δ*i*	w_i
Recapture rate (p)	**constant**			**20**	**277.97**	**326.12**	**0.00**	**0.76**
	state			22	274.44	328.46	2.33	0.24
	t			24	274.90	335.02	8.90	0.01
	state + *t*			26	274.37	340.85	14.72	0.00
Survival rate (Φ)	**constant**	**constant**		**14**	**288.93**	**320.78**	**0.00**	**0.62**
		state		18	280.67	323.19	2.41	0.19
		t		16	286.46	323.54	2.76	0.16
		state + *t*		20	277.97	326.12	5.34	0.04
Transition rate (ψ)	**constant**	**constant**	**state**	**10**	**295.53**	**317.48**	**0.00**	**0.82**
			state + *t*	14	288.93	320.78	3.30	0.16
			constant	5	315.22	325.72	8.24	0.01
			t	9	308.67	328.25	10.77	0.00

See Figure S6 for model notation. *K*: number of estimable parameters, dev: deviance, QAICc: Akaike's information criterion for small sample sizes ($QAIC_c$) correcting for overdispersion by including an estimate of model deviance (c-hat = model deviance/*df*) for the global model, Δ*i*: the QAICc difference between the current model and the model with the lowest QAICc value, w_i: Akaike weight, state: state dependent rates, constant: constant rates, *t*: time-dependent rates.

Table S8. Initial state rates, transition rates and recapture rates and ± 95% Confident Interval: CI for female Manx shearwaters. Estimates were obtained from best-supported model in Table S6.

Parameters	Capture	To	Estimates	CI-	CI+	SE
Initial state	EGG	-	0.12	0.05	0.26	0.05
	CHICK	-	0.88	0.74	0.95	0.05
	SKIP	-	0.00	0.00	0.00	0.00
Survival	-	-	0.87	0.77	0.93	0.04
Transition rate	SKIP	EGG	0.00	0.00	0.00	0.00
	SKIP	CHICK	0.79	0.36	0.96	0.16
	SKIP	SKIP	0.21	0.04	0.64	0.16
	EGG	EGG	0.08	0.01	0.39	0.07
	EGG	CHICK	0.61	0.33	0.83	0.14
	EGG	SKIP	0.31	0.11	0.61	0.13
	CHICK	SKIP	0.22	0.13	0.35	0.05
	CHICK	EGG	0.21	0.13	0.32	0.05
	CHICK	CHICK	0.57	0.45	0.69	0.06
Recapture rate	EGG	-	1.00	1.00	1.00	0.00
	CHICK	-	1.00	1.00	1.00	0.00
	SKIP	-	0.46	0.24	0.71	0.13

Table S9. Initial state rates, transition rates and recapture rates and ± 95% Confident Interval: CI for male Manx shearwaters. Estimates were obtained from the best-supported model in Table S7.

Parameters	Capture	To	Estimates	CI-	CI+	SE
Initial state	EGG	-	0.06	0.02	0.18	0.04
	CHICK	-	0.89	0.77	0.96	0.04
	SKIP	-	0.04	0.01	0.15	0.04
Survival	-	-	0.77	0.67	0.85	0.04
Transition rate	SKIP	EGG	0.24	0.03	0.75	0.21
	SKIP	CHICK	0.76	0.25	0.97	0.21
	SKIP	SKIP	0.00	0.00	0.00	0.00
	EGG	EGG	0.09	0.01	0.45	0.09
	EGG	CHICK	0.81	0.48	0.95	0.12
	EGG	SKIP	0.09	0.01	0.45	0.09
	CHICK	SKIP	0.10	0.04	0.20	0.04
	CHICK	EGG	0.26	0.16	0.38	0.06
	CHICK	CHICK	0.64	0.52	0.75	0.06
Recapture rate	EGG	-	0.94	0.80	0.98	0.04
	CHICK	-	0.94	0.80	0.98	0.04
	SKIP	-	0.94	0.80	0.98	0.04

References

1. Lecomte V.J., Sorci G., Cornet S., Jaeger A., Faivre B., Arnoux E., Gaillard M., Trouve C., Besson D., Chastel O., et al. 2010 Patterns of aging in the long-lived wandering albatross. *Proceedings of the National Academy of Sciences of the United States of America* **107**, 6370-6375.
2. McKnight A., Allyn A.J., Duffy D.C., Irons D.B. 2013 'Stepping stone' pattern in Pacific Arctic tern migration reveals the importance of upwelling areas. *Marine Ecology Progress Series* **491**, 253-+.
3. Gaston A.J., Collins B.T. 1988 The use of knock-down tags to detect changes in occupancy among burrow-nesting seabirds: What is an adequate sample size? *Canadian Wildlife Service-Progress Notes* **172**, 1-4.
4. Shoji A., Gaston A.J. 2010 Comparing methods for monitoring nest attendance in Ancient Murrelets. *Waterbirds* (33), 260-263.
5. Brooke M. 1990 *The Manx Shearwater*. London, UK, T. and A. D. Poyser.
6. Hamer K.C., Hill J.K. 1997 Nestling obesity and variability of food delivery in Manx Shearwaters, Puffinus puffinus. *Functional Ecology* **11**(4), 489-497.
7. Bonter D.N., Bridge E.S. 2011 Applications of radio frequency identification (RFID) in ornithological research: a review Aplicación de la identificación de radio frecuencia (RFID) en investigaciones ornitológicas: una revisión. *Journal of Field Ornithology* **82**(1), 1-10.
8. Riou S., Hamer K.C. 2008 Predation risk and reproductive effort: impacts of moonlight on food provisioning and chick growth in Manx shearwaters. *Animal Behaviour* **76**, 1743-1748.
9. Freund Y., Schapire R.E. 1996 Experiments with a new boosting algorithm. In *Proceedings of the Thirteenth International Conference on Machine Learning* (ed. Kaufmann M.), pp. 148–156.
10. Zhu J., Zou H., Rosset S., Hastie T. 2009 Multi-class AdaBoost. In *Statistics and its interface*, pp. 349–360.
11. Pradel R. 2005 Multievent: An extension of multistate capture-recapture models to uncertain states. *Biometrics* **61**(2), 442-447.
12. Guilford T., Meade J., Willis J., Phillips R.A., Boyle D., Roberts S., Collett M., Freeman R., Perrins C.M. 2009 Migration and stopover in a small pelagic seabird, the Manx shearwater *Puffinus puffinus*: insights from machine learning. *Proceedings of the Royal Society B-Biological Sciences* **276**(1660), 1215-1223.
13. Elliott K.H., McFarlane-Tranquilla L., Burke C.M., Hedd A., Montevecchi W.A., Anderson W.G. 2012 Year-long deployments of small geolocators increase corticosterone levels in murres. *Marine Ecology-Progress Series* **466**, 1-7.
14. Büche B., Stubbings E., Boyle D., Perrins C., Yates L. 2013 Seabird monitoring on Skomer Island in 2013. In *JNCC Contract Report*
15. Pradel R., Maurin-Bernier L., Gimenez O., Genovart M., Choquet R., Oro D. 2008 Estimation of sex-specific survival with uncertainty in sex assessment. *Canadian Journal of Statistics* **36**(1), 29-42.
16. Choquet R., Rouan L., Pradel R. 2009 “Program e-surge: A software application for fitting Multievent models. In *Modeling demographic processes in marked populations* (eds. Thomson D.L., Cooch E.G., Conroy M.J.), pp. 845-865. Dunedin, New Zealand, Springer.

17. Cormack R.M. 1964 Estimates of Survival from Sighting of Marked Animals. *Biometrika* **51**(3-4), 429-438.
18. Choquet R., Lebreton J.D., Gimenez O., Reboulet A.M., Pradel R. 2009 U-CARE: Utilities for performing goodness of fit tests and manipulating CApture-REcapture data. *Ecography* **32**(6), 1071-1074.
19. Grosbois V., Tavecchia G. 2003 Modeling dispersal with capture-recapture data: Disentangling decisions of leaving and settlement. *Ecology* **84**(5), 1225-1236.
20. Anderson D.R., Brnham K.P. 2002 Avoiding pitfalls when using information-theoretic methods. *Journal of Wildlife Management* **66**, 912-918.
